# The impact of the COVID-19 pandemic on the mental health of Rohingya refugees with pre-existing health problems in Bangladesh

**DOI:** 10.1186/s13031-022-00443-3

**Published:** 2022-03-03

**Authors:** Somen Palit, Huifang Yang, Jiangping Li, Md. Abdullah Saeed Khan, Mohammad Jahid Hasan

**Affiliations:** 1 Ningxia Medical University, Yinchuan, China; 2 Infectious Disease Hospital, Dhaka, Bangladesh; 3 Sirajul Islam Medical College and Hospital, Dhaka, Bangladesh

**Keywords:** Mental health, COVID-19, Rohingya, Refugee, Pandemic, RHS-15

## Abstract

**Background:**

Mental disorders among refugees have been well explored in several studies. However, longitudinal studies on the impact of the pandemic on refugee populations are widely lacking. This study was designed to examine the impact of the current pandemic on the mental health of Rohingya refugees living in Bangladesh.

**Method:**

This longitudinal study involved a convenience sample of 732 Rohingya people with pre-existing health problems who lived in the Kutupalong refugee camp in Cox’s Bazar, Bangladesh. The first recruitment was performed on 5 July 2019 (prepandemic visit) and assessed the health status of refugees using the Refugee Health Screener-15 (RHS-15). The follow-up survey was conducted on 10 November 2020, approximately 15 months later, during the pandemic. A total of 342 Rohingya refugees who completed the initial survey participated in the follow-up survey. A newly developed COVID-19 Impact on Quality of Life (COV19-QoL) scale was used alongside the RHS-15 scale during the second survey. Ethical measures were taken in compliance with the current Declaration of Helsinki. The analysis was performed using SPSS 26.

**Result:**

A total of 342 Rohingya refugees completed this longitudinal survey. The average age of participants was 32.25 ± 14.01 years (SD), and the predominant age group was ≤ 30 years (n = 207, 60.5%). Most of the participants were female (n = 209, 61.1%). A significant increase in stress was noted from the prepandemic to pandemic periods, as determined by the RHS-15 scale (RHS-15 Part I: 22.96 ± 8.43 vs. 46.72 ± 1.87, p < 0.001; and RHS-15 Part II: 4.43 ± 1.59 vs. 6.91 ± 1.49, p < 0.001). The mean COV19-QoL score of the participants was 4.47 ± 0.15 (out of 5), indicating a perceived negative impact of the pandemic in their lives. In the multiple regression analysis, female sex (β = 0.604, p = 0.017) and COV19-QoL score (β = 2.537, p = 0.003) were significantly associated with higher perceived distress among participants.

**Conclusion:**

Rohingya refugees experienced a significant deterioration of mental health during the COVID-19 pandemic. Alongside other socioeconomic, environmental, and political factors, the pandemic itself might have been a crucial contributor to this negative trend.

**Supplementary Information:**

The online version contains supplementary material available at 10.1186/s13031-022-00443-3.

## Background

According to the 1951 Refugee Convention, refugees are individuals who have been forcibly displaced outside their native countries [1]. Currently, there are approximately 26 million refugees around the world [2]. Among them, over 1.1 million forcibly displaced Myanmar nationals known as Rohingya have been accommodated in Bangladesh, including 877,710 Rohingya people sheltering in the world’s largest refugee camp in Cox’s Bazar, a district in southeastern Bangladesh [3, 4].

Forced migration exerts short- and long-term effects on refugees’ mental health, including anxiety disorders, depression, posttraumatic stress disorders (PTSD), emotional distress, and other effects [5, 6]. The incidence varies with geographical location, but the overall rates ranged between 27 and 33.1% for clinical depression, 3.8–16.7% for anxiety disorders, and 14–20.5% for PTSD [7–9]. The incidence varies with timelines as well. An approximately 4% increase in depression, a 2% increase in anxiety disorder, and a 1% decrease in PTSD were observed among Syrian refugees over a 1.5-year period after they fled from their country [9]. Among the Rohingya people, approximately 89%, 84%, and 61% suffer from depression, emotional distress, and PTSD, respectively [10].

Several factors were associated with their mental distress. Trauma, violence, and restrictions in education and freedom of movement were the prime reasons. The lack of appropriate health care facilities and inadequate social facilities such as accommodations, along with loneliness and boredom after arrival in Bangladesh, are other sources of stress [10–12]. More recently, the pandemic caused by coronavirus disease 2019 (COVID-19) has been considered another deleterious factor contributing to poor mental health. The pandemic gave rise to anxiety and depression in the people of Bangladesh [13] and in other populations in countries around the world [14–16]. However, the effects of COVID-19 among vulnerable Rohingya refugees, particularly the effects on their mental health, remain underexplored and call for an evaluation. Therefore, the study aims to determine the impact of the COVID-19 pandemic on the mental health of Rohingya refugees in Bangladesh.

## Methods

### Design and hypotheses

A longitudinal study design was used to evaluate emotional distress and mental health in Rohingya refugees. The first survey began on 5 July 2019 and was scheduled to end after three months. The initial plan was to follow up with participants after one year. However, the follow-up survey could not be carried out until 10 November 2020, 15 months after the baseline survey. The pandemic started in March 2020 [13] in Bangladesh and was responsible for more than one million confirmed cases of COVID-19, with thousands of deaths in the country [17]. The total number of COVID-19 cases among the Rohingya population was more than 400, with ten reported deaths as of March 2021 [18]. Amidst the pandemic, we conducted the second survey, and we hypothesized that the ongoing pandemic could affect the mental health of Rohingya refugees in addition to their usual stress and suffering. To examine the effect of the pandemic among this population, we used a COVID-19-specific quality of life scale [19] during the second survey in addition to the *Refugee Health Screener-15* (RHS-15).

### Sampling frame, sampling, and data collection procedure

The sampling frame was a cohort of refugees 18 years or older who were forcibly displaced from Myanmar during the 2017 mass killing incidents in Rakhine State and who resettled in the Kutupalong camp of the Cox’s Bazar district in Bangladesh. These refugees received their name ‘Rohingya’ from the local colloquial transformation of their original designation “*rui hang gya”* in Burmese. Among the refugees, those who attended the health care center in the camp due to different health problems were primarily targeted for inclusion in the study. All patients attending the study center during the first survey were conveniently sampled (N = 732) and assessed using a pretested and structured questionnaire. A team of 20 Burmese migrant volunteers who were living around the refugee camp and who understood Bangla (Bengali) and the Rohingya dialect were trained using the questionnaire. They conducted face-to-face interviews with participants in their language and entered the information into the translated Bangla questionnaire (please see "The *refugee health screener-15* (RHS-15)" section). The interview took 10–15 min to complete. Participants who had a past history of mental health disorders or a history of receiving drugs for any particular mental health problem were screened and not included in the study. Written informed consent was obtained from all subjects before participation. The consent form was explained to the participants by the volunteers in their dialect. A total of 732 responses were collected during the first survey. However, only 342 participants from the initial sample completed the final survey. (Details of the study participant recruitment are available in Additional file 1: Figure S1.) Participants who were suspected of having suicidal thoughts or major psychiatric disorders were referred to the mental health and psychosocial support center situated in Kutupalong, accompanied by one of the volunteers of the study.

### Instruments

The surveys consisted of questions related to demographic profiles, important medical history, and health screening tools. The Refugee Health Screener 15 (RHS-15) was used during the first and follow-up surveys. The COVID-19-Impact on Quality of Life (COV19-QoL) scale v 1.5 [19] was used as an additional instrument to assess the impact of COVID-19 among participants during the follow-up survey.

### The *refugee health screener-15* (RHS-15)

The RHS-15 is a screening tool used to assess emotional distress and mental health among recently settled refugees. The RHS-15 has 13 symptom items, one coping item, and one distress thermometer (DT). The instructions for the symptom items are to “indicate the degree to which the symptom has been bothersome to any individual over the past month.” Possible responses are 0 = not at all, 1 = a little bit, 2 = moderately, 3 = quite a bit, and 4 = extremely. To compensate for literacy and cultural barriers in the understanding of the scales, symbols of jars with beans were used to obtain possible responses, where variable amounts of beans were relevant to each response. The coping item assesses beliefs about the general ability to cope with stress. Responses range from 0 (able to handle or cope with anything that comes your way) to 4 (unable to handle or cope with anything). The DT looks like a thermometer, with a “0” (“no distress—things are good”) at the bottom and a “10” (“extreme distress—I feel as bad as I ever have”) at the top. The 13 symptom and 1 coping item responses are added to obtain a 14-item total score. According to the currently recommended scoring, an “RHS-15 case” is defined as a total score of 12 or greater or a DT of 5 or greater, which is supported by previous post hoc testing [20]. The RHS-15 questions 1–14 showed acceptable internal consistency among our participants (Cronbach’s α = 0.735).

#### COVID-19–impact on quality of life (COV19-QoL) scale v 1.5 [19]

The scale was developed to serve as a tool for assessing the impact of the COVID-19 pandemic in general on mental health, reflecting participants’ feelings and thoughts during the past seven days, and findings have been reported in various studies on mental health. We adopted this scale to examine the impact of the pandemic on the Rohingya population. It consists of 6 questions rated on a 5-point Likert scale, where “Completely disagree” denotes a score of one and “Completely agree” has a score of 5. Scores are analyzed separately for each item. Then, the scores of all items are summed and divided by the number of items (i.e., 6). Hence, the total score will be the average of all the items. A higher score indicates greater impact.

### Translation of instruments

Neither of the two tools had versions validated in Bengali; therefore, the interviewer translated them from English to our local language (Bangla) with the best possible effort to maintain the original meaning and context. Another independent translator, unaware of the original study, then translated the Bangla version back into English. Next, the two versions were matched, and any discrepancy in the wordings of the Bangla version was corrected. Then, the volunteers were trained on the questionnaire. As the volunteers could speak the Rohingya dialect, the questionnaire was rehearsed in that language to ensure the correct recording of the information. After that, the volunteers went to the field, interviewed the Rohingya refugees in a language understandable to them, and recorded their responses in the Bangla questionnaire. However, many of the Rohingya refugees understood Bangla, particularly in the follow-up interviews.

### Statistical analyses

Statistical analyses were conducted using IBM SPSS 26. Missing values were managed by subtracting the data from the final dataset. No mean imputation was used. Continuous variables are expressed as the mean ± standard deviation, whereas categorical data are expressed as the frequency or percentage. Student’s t test and the chi-square test were used when applicable. Stepwise linear regression was run to investigate the effect of COVID-19 on the mean change in the RHS-15 score among the Rohingya refugees. The results were expressed with 95% confidence intervals (CIs). P values less than 0.05 were considered to indicate statistical significance.

## Results

### Demographics of the study population at the prepandemic visit

The average age of the 342 participants (who completed both surveys) was 32.25 ± 14.01 years (SD), with the majority being aged ≤ 30 years (n = 207, 60.5%). Most of the participants were female (n = 209, 61.1%) and married (n = 248, 72.5%). Among all of them, 103 participants had hypertension [HTN] (30.1%), 42 had ischemic heart disease [IHD] (12.3%), 31 had bronchial asthma [BA] (9.1%), 28 had chronic obstructive pulmonary disease [COPD] (8.2%) and 20 had diabetes mellitus [BA] (5.8%) (Table 1). The baseline characteristics of the 732 participants are described in Additional file 1: Table S1. A comparison of the demographic and comorbidity characteristics of participants who completed the follow-up survey (n = 342) and those who did not (n = 390) revealed statistical similarity except for bronchial asthma and the prepandemic RHS-15 Parts I and II scores (Additional file 1: Table S2). The prepandemic RHS-15 scores were significantly higher among participants who were lost to follow-up.

**Table 1:** Participants characteristics at pre-pandemic visit (n = 342)

Variable	Frequency	Percentage (%)
Age (years)		
Mean ± SD	32.25 ± 14.01	
Median (min–max)	27 (18–90)	
Age category		
≤ 30	207	60.5
31–40	57	16.7
41–50	39	11.4
51–60	25	7.3
> 60	14	4.1
Sex		
Female	209	61.1
Male	133	38.9
Marital status		
Married	248	72.5
Unmarried	68	19.9
Divorced	20	5.8
Widow	6	1.8
Comorbidities		
HTN	103	30.1
IHD	42	12.3
BA	31	9.1
COPD	28	8.2
DM	20	5.8

HTN: Hypertension; BA: Bronchial Asthma; IHD: Ischemic Heart Disease; COPD: Chronic Obstructive Pulmonary Disease; DM: Diabetes Mellitus

### Prepandemic emotional distress and mental health assessment of the Rohingya population (at first survey)

Table 2 shows the prepandemic and pandemic RHS-15 Part I (Items 1–14) and part II (distress thermometer) scores among the participants. The average total scores of RHS-15 Part I and Part II at the prepandemic visit were 22.96 ± 8.43 and 4.42 ± 1.59, respectively. Participants who were aged > 30 years had significantly higher RHS-15 Part I scores than those aged ≤ 30 years (p < 0.001). In addition, married participants had a significantly higher RHS-15 Part I score than those who were single (p < 0.05). The score was also significantly higher among those with IHD (p < 0.05). However, the Part II score was statistically similar across different participant characteristics (p > 0.05).

**Table 2:** RHS Scores at baseline and follow up assessment of the participants

Variable	Pre-pandemic assessment (N = 342)				During pandemic assessment (N = 342)			
	RHS-15 Part I^a^	p-value^*^	RHS-15 Part II^a^	p-value*	RHS-15 Part I^a^	p-value	RHS-15 Part II^a^	p-value*
Total score								
Mean ± SD	22.96 ± 8.43		4.42 ± 1.59		46.72 ± 1.87		6.91 ± 1.49	
Median (Min–Max)	23 (2–44)		5 (0–10)		47 (40–51)		7 (4–10)	
Age category (years)								
≤ 30	21.94 ± 8.34	0.005	4.43 ± 1.59	0.980	46.57 ± 1.84	0.052	6.89 ± 1.47	0.766
> 30	24 .53 ± 8.35		4.43 ± 1.59		46.97 ± 1.88		6.94 ± 1.51	
Gender								
Female	22.71 ± 8.23	0.486	4.51 ± 1.54	0.244	46.89 ± 1.74	0.033	7.28 ± 1.40	< 0.001
Male	23.36 ± 8.74		4.30 ± 1.67		46.45 ± 2.02		6.33 ± 1.44	
Marital status								
Married	23.38 ± 8.29	0.134	4.48 ± 1.47	0.320	46.76 ± 1.83	0.585	6.91 ± 1.51	0.984
Single^b^	21.85 ± 8.71		4.28 ± 1.88		46.64 ± 1.96		6.91 ± 1.45	
Comorbidities								
HTN								
Present	21.66 ± 8.28	0.061	4.42 ± 1.39	0.943	46.63 ± 2.04	0.529	7.15 ± 0.14	0.057
Absent	23.52 ± 8.44		4.43 ± 1.68		46.77 ± 1.79		6.81 ± 1.51	
BA								
Present	22.65 ± 8.91	0.827	4.06 ± 1.99	0.185	47.00 ± 1.48	0.396	6.74 ± 1.59	0.505
Absent	22.99 ± 8.39		4.46 ± 1.55		46.70 ± 1.87		6.92 ± 1.48	
IHD								
Present	25.40 ± 6.94	0.021	4.24 ± 1.72	0.414	46.30 ± 2.21	0.121	6.91 ± 1.51	0.486
Absent	22.62 ± 8.57		4.45 ± 1.58		46.79 ± 1.81		6.93 ± 1.51	
COPD								
Present	23.14 ± 7.71	0.906	4.57 ± 1.91	0.618	46.74 ± 1.88	0.801	6.43 ± 1.69	0.073
Absent	22.95 ± 8.49		4.41 ± 1.57		46.64 ± 1.70		6.96 ± 1.47	
DM								
Present	26.70 ± 4.90	0.003	4.50 ± 0.82	0.709	47.40 ± 1.14	0.097	7.90 ± 0.96	< 0.001
Absent	22.73 ± 8.56		4.42 ± 1.63		46.69 ± 1.89		6.85 ± 1.49	

RHS- Refugee Health Screener; HTN: Hypertension; BA: Bronchial Asthma; IHD: Ischemic Heart Disease; COPD: Chronic Obstructive Pulmonary Disease; DM: Diabetes Mellitus

^a^RHS-15 part I consists of items 1–14 and part II consists of item 15 (distress thermometer)

^b^Including unmarried, divorced and widowed

^*^p value estimated by independent sample t-test

p value < 0.05 considered statistically significant

### Assessment of a similar population during the pandemic (follow-up visit at 15 months)

At the follow-up assessment, the mean RHS-15 Part I and Part II scores were 46.72 ± 1.87 and 6.91 ± 1.49, respectively. The Part I and Part II scores were significantly higher among females than among males (p < 0.05). Additionally, the Part II score was significantly higher among diabetic participants than among nondiabetic participants (p < 0.001). A comparison of RHS-15 Part I and Part II scores across different characteristics of all participants who completed the survey at baseline revealed a significantly higher Part I score in participants who were aged ≤ 30 years, who were married, and who had BA, IHD, or DM (p < 0.05 for all) (Additional file 1: Table S3).

There was a significant increase in the average scores of both parts of the RHS-15 from the initial to follow-up assessments. The increment was substantial across all sociodemographic and comorbidity categories (Table 3). Among all participants who completed the follow-up (n = 342), the average increases in RHS-15 Part I and Part II scores were 23.77 ± 8.78 and 2.48 ± 2.28, respectively (Additional file 1: Table S4). However, the mean change in the Part I score was significantly lower in the > 30 age group in the presence of IHD and DM (p < 0.05). Moreover, the mean change in the Part II score was significantly higher among female and diabetic participants (p < 0.05).

**Table 3:** Comparison of initial and follow-up RHS scores of participants before and after commencement of COVID-19 pandemic (n = 342)

Variable	RHS-15 Part I^a^		p-value*	RHS-15 Part II^a^		p-value*
	Pre-pandemic visit	During pandemic visit		Pre-pandemic visit	During pandemic visit	
Total score	22.96 ± 8.43	46.72 ± 1.87	< 0.001	4.43 ± 1.59	6.91 ± 1.49	< 0.001
Age category						
≤ 30	21.94 ± 8.33	46.57 ± 1.84	< 0.001	4.42 ± 1.59	6.89 ± 1.47	< 0.001
> 30	24.53 ± 8.35	46.97 ± 1.88	< 0.001	4.43 ± 1.59	6.94 ± 1.52	< 0.001
Sex						
Female	22.71 ± 8.23	46.89 ± 1.74	< 0.001	4.51 ± 1.54	7.28 ± 1.40	< 0.001
Male	23.36 ± 8.74	46.45 ± 2.02	< 0.001	4.30 ± 1.67	6.30 ± 1.44	< 0.001
Marital status						
Married	23.38 ± 8.29	46.76 ± 1.83	< 0.001	4.48 ± 1.48	6.91 ± 1.51	< 0.001
Single^b^	21.85 ± 8.71	46.63 ± 1.95	< 0.001	4.29 ± 1.87	6.91 ± 1.45	< 0.001
HTN						
Present	21.66 ± 8.29	46.63 ± 2.04	< 0.001	4.42 ± 1.38	7.15 ± 1.42	< 0.001
Absent	23.52 ± 8.44	46.77 ± 1.79	< 0.001	4.43 ± 1.68	6.81 ± 1.51	< 0.001
BA						
Present	22.65 ± 8.91	47.00 ± 1.73	< 0.001	4.06 ± 1.99	6.74 ± 1.59	< 0.001
Absent	22.99 ± 8.39	46.70 ± 1.87	< 0.001	4.46 ± 1.55	6.93 ± 1.48	< 0.001
IHD						
Present	25.40 ± 6.94	46.31 ± 2.21	< 0.001	4.24 ± 1.72	6.79 ± 1.37	< 0.001
Absent	22.62 ± 8.57	46.79 ± 1.81	< 0.001	4.45 ± 1.58	6.93 ± 1.51	< 0.001
COPD						
Present	23.14 ± 7.71	46.64 ± 1.70	< 0.001	4.57 ± 1.91	6.43 ± 1.69	< 0.001
Absent	22.95 ± 8.49	46.74 ± 1.88	< 0.001	4.41 ± 1.57	6.95 ± 1.47	< 0.001
DM						
Present	26.70 ± 4.90	47.40 ± 1.14	< 0.001	4.50 ± 0.82	7.90 ± 0.96	< 0.001
Absent	22.73 ± 8.56	46.69 ± 1.89	< 0.001	4.42 ± 1.63	6.85 ± 1.49	< 0.001

RHS- Refugee Health Screener; HTN: Hypertension; BA: Bronchial Asthma; IHD: Ischemic Heart Disease; COPD: Chronic Obstructive Pulmonary Disease; DM: Diabetes Mellitus

^a^RHS-15 part I consists of items 1–14 and part II consists of item 15

^b^Including unmarried, divorced and widowed

^*^p value estimated by paired sample t-test

p value < 0.05 considered statistically significant

**Fig. 1:**
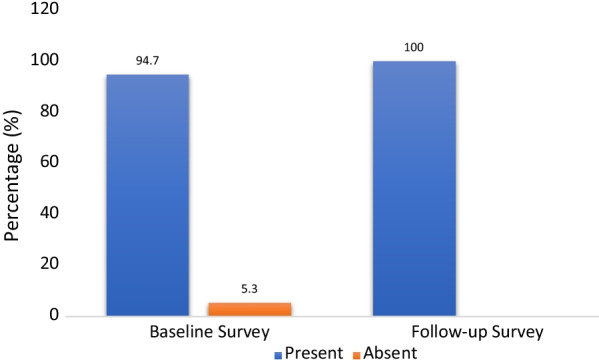
Proportion of participants positive for emotional distress at pre-pandemic (base-line survey) and during pandemic (follow-up) assessment (n = 342). (A person is said to screen positive for emotional distress in RHS-15 scale if she/he has a score of ≥ 12 in question 1–14 OR ≥ 5 in distress thermometer)

At the baseline assessment, 94.7% of the 342 participants screened positive for psychological distress as defined by a total score ≥ 12 on questions 1–14 (RHS-15 Part I) OR ≥ 5 on the distress thermometer (RHS-15 Part II). At the follow-up assessment, all participants had developed psychological distress (Fig. 1).

### Effect of the current pandemic on mental health among Rohingya refugees

The average COV19-QoL score of the participants was 4.47 ± 0.15. The score was significantly higher in females than in males (4.49 ± 0.11 vs. 4.44 ± 0.19, p < 0.05) (Table 4). A detailed pattern of responses to the six statements of the COVID-19-QoL scale is shown in Additional file 1: Table S5. Almost all participants either agreed or strongly agreed with the statements. However, the participants were mainly worried about their personal safety (61.4% showed strong agreement). The second strongest response was associated with their increased feeling of being tense (56.4% showed strong agreement). We built a multivariate linear regression model using the change in ‘distress thermometer’ score between baseline and follow-up assessments as the dependent variable and age, sex, marital status, HTN, BA, IHD, COPD, DM and COV19-QoL score as independent variables. As the analysis was run using the stepwise method, the final model only included significant predictors, which in this case were the COV19-QoL score and the sex of the participants (Table 5). A one-unit increase in COV19-QoL score was associated with a 2.537-unit increase in distress thermometer score when participants’ sex remained constant (p < 0.05). Being female was associated with a 0.604-unit increase in distress thermometer score when participants’ COV19-QoL score remained constant (p < 0.05).

**Table 4:** COV19-QoL scale scores among participants during the pandemic visit (n = 342)

Variable	COV19-QoL^a^	p-value*
Total score		
Mean ± SD	4.47 ± 0.15	
Median (min–max)	4.5 (3.83–5.00)	
Age category		
≤ 30	4.47 ± 0.15	0.541
> 30	4.47 ± 0.14	
Sex		
Female	4.49 ± 0.11	0.001
Male	4.44 ± 0.19	
Marital status		
Married	4.48 ± 0.14	0.893
Single^b^	4.47 ± 0.15	
HTN		
Present	4.47 ± 0.15	0.724
Absent	4.48 ± 0.15	
BA		
Present	4.47 ± 0.15	0.768
Absent	4.48 ± 0.15	
IHD		
Present	4.44 ± 0.15	0.147
Absent	4.48 ± 0.15	
COPD		
Present	4.45 ± 0.19	0.392
Absent	4.48 ± 0.14	
DM		
Present	4.48 ± 0.05	0.797
Absent	4.47 ± 0.15	

HTN: Hypertension; BA: Bronchial Asthma; IHD: Ischemic Heart Disease; COPD: Chronic Obstructive Pulmonary Disease; DM: Diabetes Mellitus

^a^Average of 5-point 6 item scores

^b^Including unmarried, divorced and widowed

^*^p value estimated by independent sample t-test

p value < 0.05 considered statistically significant

**Table 5:** Linear regression model^a^ for predicting changes in distress thermometer between pre-pandemic and pandemic assessment (n = 342)

Dependent variable	Independent variable^b^	β-coefficient	Standard error	P-value	Adjusted R square
Changes in RHS-15 part-II^c^ score	Constant	− 8.028	3.840	0.037	0.045
	COV19-QoL score	2.537	0.840	0.003	
	Sex (female)	0.604	0.252	0.017	

^a^Multivariate linear regression model using stepwise method

^b^Variables excluded from the final model are: age, marital status, HTN, BA, IHD, COPD and DM

^c^RHS-15 part II is comprised of the ‘distress thermometer’ (item no 15), which measures the subjective experience of distress among participants in the past week including the day of interview

## Discussion

In 2018, the International Organization for Migration conducted a rapid mental health assessment of Rohingya refugees living in the Cox’s Bazar district of Bangladesh and reported that they were going through a substantial mental health crisis with frequent reports of feeling always sad (74%), always tense (64%) and always anxious (25%) [21]. Subsequently, another study by Riley et al. [22] in the same year reported mental health symptoms indicative of PTSD in 61% and emotional distress in 84% of participants. Mental distress was expected to wane slowly with time as the support of national and international communities was growing and people were adapting to the new environment. Hence, we initially attempted to assess the mental health status of Rohingya refugees using the RHS-15 scale at the end of 2019. However, the COVID-19 pandemic began unexpectedly and added to the existing humanitarian crisis. Hence, we extended our objective to carry out a follow-up assessment of the mental health of the already enrolled participants using the same scale. We also assessed the quality of life (QoL) of the participants using a newly developed QoL scale designed to assess the impact of COVID-19.

Our analysis revealed a significant increase in mental distress among Rohingya refugees after the onset of the COVID-19 pandemic. Moreover, the pandemic strongly impacted the QoL of the refugees and contributed significantly to the deterioration of their mental health. These findings are consistent with expectations, as COVID-19 pandemic-related lockdowns affected the mental health of people in general [23] and that of health care workers, noninfectious chronic disease patients, COVID-19 patients, and quarantined persons in particular [14]. As the Rohingya refugees were already living in a protracted environment with a scarcity of basic human requirements in some places [24], the situation worsened with the added burden of lockdowns and the disease itself.

We noted that Rohingya women were affected significantly more than men. A possible reason could be that most of the Rohingya families are headed by women, particularly because when the Myanmar army-led savagery started, most women and children fled to Bangladesh [25]. As the head of the family, the women had to endure the most stress to ensure the basic survival of the family members. They already had limited access to critical and life-saving services such as food, drinkable water, and shelter [26]. The added burden of COVID-19 worsened the existing major humanitarian disaster.

Although excluded from the final regression model, we found an association of some other factors with the mental health of Rohingya refugees in the univariate analysis. At baseline, we noted higher emotional distress among participants who were adults > 30 years compared to young adults (≤ 30 years), those who were married compared to those who were single, and those who had BA, IHD or DM (when all participants were considered). A study of the demography of Rohingya refugees suggests that the majority of the Rohingya population living in camps in Bangladesh are young (< 20 years) [27]. Another study conducted among Syrian refugees in Jordan [28] found that young people had higher coping abilities and problem-solving strategies and were free from illness. These observations might explain our findings. Additionally, married participants had to bear the responsibility of themselves and their family members, exposing them to higher stress.

Riza et al. [29] studied the determinants of refugee and migrant health in 10 European countries. They observed that the presence of chronic disease or mental health disorders was associated with worse general health among refugees. Mikolajc et al*.* [30] showed that chronic disease is associated with reduced quality of life in refugees who live in camps. Our findings at baseline and follow-up assessments of Rohingya refugees conform to these observations. However, as participants who were lost to follow-up had a significantly higher RHS-15 score (Parts I and II) at baseline, it can be argued that a significant mental health impact among these lost participants could have been missed from the study. Nonetheless, even if that were the case, it would have strengthened our current finding of worsening stress among the Rohingya refugees.

We noted increased distress among participants with one or more chronic diseases. In particular, diabetes was associated with higher distress at both baseline and follow-up evaluations. The additional challenge of seeking care for chronic diseases in addition to an existing struggle for basic needs might have negatively influenced the mental health of Rohingya refugees in Bangladesh. In addition, several other environmental, socioeconomic, and political factors could have contributed to this increased distress. The worsening security situation in the camps, fires, storms, the destruction of Rohingya shops in the camps, the government’s decision and action to relocate refugees to Bhasan Char, less funding for refugees, and decreased service provision are some of the important factors contributing to increased stress among the Rohingya people [31]. Moreover, the recent military coup [32] in Myanmar has increased the uncertainty of their return to their home country, adding to the existing anxiety and stress. Hence, the COVID-19 pandemic could be considered only one element of the multifaceted stressors that Rohingya refugees in Bangladesh are currently experiencing.

Our study findings emphasize the importance of addressing the mental health of Rohingya refugees in the context of the COVID-19 pandemic. National and international organizations working on the rehabilitation of the Rohingya people should focus on ensuring their basic needs and supporting emotional and mental health.

## Limitations and strengths of the study

This study had several limitations. It was conducted in a single camp. Participants were selected conveniently from those with pre-existing health problems. Therefore, the results might not be generalizable. More than half of the initial participants were lost to follow-up. Hence, the association of COVID-19’s impact on the changes in distress scores found in this study should be interpreted with caution. Another limitation was the use of an unvalidated translation of the instrument in Bangla. We also could not assess all the possible contributors to increased stress among this population. However, our study’s strength is that this was the first attempt to follow up on the health status of the Rohingya people before and after the start of the COVID-19 pandemic, providing essential insights into their coping amidst this added burden of unprecedented restrictions.

## Conclusion

There was considerable mental health distress among the Rohingya refugees, which increased significantly during the COVID-19 pandemic. The pandemic could be one of the significant contributors to their increasing stress over recent years.

## Supplementary Information

**Additional file 1. Figure 1**. Patient selection flow chart.** Table 1**. Sociodemographic profile and comorbidities of the participants (n = 732).** Table 2**. Comparison of characteristics between participants who completed follow-up survey and who were lost from it.** Table 3**. RHS Scores at baseline assessment of the participants (n = 732).** Table 4**. Mean changes in RHS-15 score between baseline and follow-up evaluation of participants (n = 342).** Table 5**. Participants response to COVID-19 – Impact on Quality of Life (COV19-QoL) scale.

## Data Availability

The data and material are available upon reasonable request to the corresponding author (dr.jahid61@gmail.com).

## References

[1] United Nations High Commissioner for Refugees. Convention and protocol relating to the status of refugees; 1951, p. 14. 12344222

[2] The world’s refugees in numbers: the global solidarity crisis. Amnesty international. https://www.amnesty.org/en/what-we-do/refugees-asylum-seekers-and-migrants/global-refugee-crisis-statistics-and-facts/. Accesses 18 Mar 2021

[3] Islam MM, Yunus MY (2020). Rohingya refugees at high risk of COVID-19 in Bangladesh. Lancet Glob Heal.

[4] UNHCR. Joint Government of Bangladesh—UNHCR Population Factsheet. https://data2.unhcr.org/en/documents/download/74676. Accessed 18 Mar 2021

[5] Khan S, Haque S (2021). Trauma, mental health, and everyday functioning among Rohingya refugee people living in short- and long-term resettlements. Soc Psychiatry Psychiatr Epidemiol.

[6] Mollica RF (2001). Longitudinal study of psychiatric symptoms, disability, mortality, and emigration among Bosnian refugees. JAMA.

[7] Wulfes N, del Pozo MA, Buhr-Riehm B, Heinrichs N, Kröger C (2019). Screening for posttraumatic stress disorder in refugees: comparison of the diagnostic efficiency of two self-rating measures of posttraumatic stress disorder. J Trauma Stress.

[8] Richter K, Peter L, Lehfeld H, Zäske H, Brar-Reissinger S, Niklewski G (2018). Prevalence of psychiatric diagnoses in asylum seekers with follow-up. BMC Psychiatry.

[9] Borho A, Viazminsky A, Morawa E, Schmitt GM, Georgiadou E, Erim Y (2020). The prevalence and risk factors for mental distress among Syrian refugees in Germany: a register-based follow-up study. BMC Psychiatry.

[10] Fortify Rights. “The Torture in My Mind”: The right to mental health for Rohingya survivors of genocide in Myanmar and Bangladesh. J Chem Inf Model. 2020;1–102

[11] Porter M, Haslam N (2005). Predisplacement and postdisplacement factors associated with mental health of refugees and internally displaced persons: a meta-analysis. J Am Med Assoc.

[12] Jannesari S, Hatch S, Prina M, Oram S (2020). Post-migration social-environmental factors associated with mental health problems among asylum seekers: a systematic review. J Immigr Minor Heal.

[13] Hasan MJ, Tabssum T, Ambia NE, Zaman MS, Rahman M, Khan AS (2021). Mental health of the COVID-19 patients in Bangladesh. Mymensingh Med J.

[14] Wu T, Jia X, Shi H, Niu J, Yin X, Xie J (2021). Prevalence of mental health problems during the COVID-19 pandemic: a systematic review and meta-analysis. J Affect Disord.

[15] Vindegaard N, Benros ME (2020). COVID-19 pandemic and mental health consequences: systematic review of the current evidence. Brain Behav Immun.

[16] Rajkumar RP (2020). COVID-19 and mental health: a review of the existing literature. Asian J Psychiatr.

[17] Coronavirus cases: Worldometers. https://www.worldometers.info/coronavirus/country/bangladesh/. Accessed 20 July 2021

[18] Rohingya Crisis Situation Report #4. World Health Organization. https://cdn.who.int/media/docs/default-source/searo/bangladesh/bangladesh---rohingya-crisis---pdf-reports/sitreps/2021/who-cxb-situation-report-4.pdf?sfvrsn=2497b180_15. Accessed 17 Mar 2021.

[19] Repišti S, Jovanović N, Kuzman MR, Medved S, Jerotić S, Ribić E (2020). How to measure the impact of the COVID-19 pandemic on quality of life: COV19-QoL—the development, reliability and validity of a new scale. Glob Psychiatry..

[20] Hollifield M, Verbillis-Kolp S, Farmer B, Toolson EC, Woldehaimanot T, Yamazaki J (2013). The Refugee Health Screener-15 (RHS-15): development and validation of an instrument for anxiety, depression, and PTSD in refugees. Gen Hosp Psychiatry.

[21] International Organisation for Migration. Rapid mental health and psychosocial support needs of the Rohingya refugees displaced in Cox’s Bazar. 2018. https://www.iom.int/sites/default/files/our_work/DMM/Migration-Health/final_report_march_2018_-iom_mhd.pdf

[22] Riley A, Akther Y, Noor M, Ali R, Welton-Mitchell C (2020). Systematic human rights violations, traumatic events, daily stressors and mental health of Rohingya refugees in Bangladesh. Confl Health.

[23] Prati G, Mancini AD. The psychological impact of COVID-19 pandemic lockdowns: a review and meta-analysis of longitudinal studies and natural experiments. Psychol Med. 2021;8–11. 10.1017/S0033291721000015 PMC7844215 33436130

[24] Banerjee S (2019). The Rohingya crisis: a health situation analysis of refugee camps in Bangladesh. Obs Res Found.

[25] Karin S, Chowdhury MA, Shamim I (2020). Status of Rohingya refugees in Bangladesh: a comparative study with emphasis on aspects of women and girls in camps of Kutupalong, Cox’s Bazar, Bangladesh. OALib.

[26] Banik R, Rahman M, Hossain MM, Sikder MT, Gozal D (2020). COVID-19 pandemic and Rohingya refugees in Bangladesh: What are the major concerns?. Glob Public Health.

[27] Chowdhury MAK, Billah SM, Karim F, Khan AN, Islam S, Arifeen S El. Report on demographic profiling and needs assessment of maternal and child health (MCH) care for the Rohingya refugee population in Cox’s Bazar, Bangladesh; 2018. http://dspace.icddrb.org/jspui/handle/123456789/9067

[28] Alzoubi FA, Al-Smadi AM, Gougazeh YM (2019). Coping strategies used by syrian refugees in Jordan. Clin Nurs Res.

[29] Riza E, Karnaki P, Gil-Salmerón A, Zota K, Ho M, Petropoulou M (2020). Determinants of refugee and migrant health status in 10 European Countries: the Mig-healthcare project. Int J Environ Res Public Health.

[30] Mikolajczyk RT, Maxwell AE, Eljedi A. Quality of Life and Chronic Illness among Refugee Populations. In: Handbook of Disease Burdens and Quality of Life Measures. New York: Springer New York; 2010. pp. 3397–412. https://link.springer.com/referenceworkentry/10.1007%2F978-0-387-78665-0_196

[31] Sullivan DP. Fading Humanitarianism: The Dangerous Trajectory of the Rohingya Refugee Response in Bangladesh’, *Refugees International*, (May); 2021. https://reliefweb.int/report/bangladesh/fading-humanitarianism-dangerous-trajectory-rohingya-refugee-response-bangladesh.

[32] Cuddy A. *Myanmar coup: What is happening and why?*, *BBC*; 2021. https://www.bbc.com/news/world-asia-55902070. Accessed 29 Nov 2021.

