# Supplementary material for: The impact of the COVID-19 pandemic on the mental health of Rohingya refugees with pre-existing health problems in Bangladesh
Source: Confl Health. 2022 Mar 3;16:10. doi: 10.1186/s13031-022-00443-3 (PMC8892402; doi:10.1186/s13031-022-00443-3)
Supplement: Supplementary file 1 — Additional file 1. Figure 1. Patient selection flow chart. Table 1. Sociodemographic profile and comorbidities of the participants (n = 732). Table 2. Comparison of characteristics between participants who completed follow-up survey and who were lost from it. Table 3. RHS Scores at baseline assessment of the participants (n = 732). Table 4. Mean changes in RHS-15 score between baseline and follow-up evaluation of participants (n = 342). Table 5. Participants response to COVID-19 – Impact on Quality of Life (COV19-QoL) scale. [file 13031_2022_443_MOESM1_ESM.docx]

Number of approached Rohingya Refugees
(n = 998 )

Case excluded (n=251)

(n=10) Didn’t agree to participate

(n=198) Under-age

(n=30) Pregnancy and lactation

(n=3) Critically ill

(n=10) Due to major psychiatric illness

Final enrollment for initial assessment (n = 747)

Number of participants with complete data at initial assessment for analysis
(n = 732)

Incomplete Data (n=15)

Final enrollment in follow-up assessment
(n = 423)

Lost from follow-up (n=309)

Number of participants with complete data at follow-up for analysis
(n = 342)

Incomplete Data (n=81)

**Supplementary figure 1. Patient selection flow chart**

**Supplementary** **Table 1. Sociodemographic profile and comorbidities of the participants (n=732)**

| **Variable** | **Frequency** | **Percentage (%)** |
| --- | --- | --- |
| **Age (years)** |  |  |
| Mean ±SD | 32.65 ±14.54 |  |
| Median (Min-Max) | 27 (18 – 90) |  |
| **Age category** |  |  |
| ≤ 30 | 440 | 60.1 |
| 31 – 40 | 110 | 15.0 |
| 41 – 50 | 84 | 11.5 |
| 51 – 60 | 65 | 8.9 |
| > 60 | 33 | 4.5 |
| **Sex** |  |  |
| Female | 425 | 58.1 |
| Male | 307 | 41.9 |
| **Marital Status** |  |  |
| Married | 533 | 72.8 |
| Unmarried | 147 | 20.1 |
| Divorced | 37 | 5.1 |
| Widow | 15 | 2.0 |
| **Comorbidities** |  |  |
| HTN | 196 | 26.8 |
| BA | 139 | 19.0 |
| IHD | 97 | 13.3 |
| COPD | 56 | 7.7 |
| DM | 38 | 5.2 |

HTN: Hypertension; BA: Bronchial Asthma; IHD: Ischemic Heart Disease; COPD: Chronic Obstructive Pulmonary Disease; DM: Diabetes Mellitus

**Supplementary Table 2. Comparison of characteristics between participants who completed follow-up survey and who were lost from it**

| **Variable** | **Complete follow-up**  **(n=342)**  **n(%)** | **Lost from follow-up**  **(n=390)**  **n(%)** | **p-value** |
| --- | --- | --- | --- |
| **Age (years)** |  |  |  |
| Mean±SD | 32.25 ±14.01 | 33.00 ±15.01 | 0.497 |
| **Age category** |  |  |  |
| ≤ 30 | 207 (60.5) | 233 (59.7) | 0.526 |
| 31 – 40 | 57 (16.7) | 53 (13.6) |  |
| 41 – 50 | 39 (11.4) | 45 (11.5) |  |
| 51 – 60 | 25 (7.3) | 40 (10.3) |  |
| > 60 | 14 (4.1) | 19 (4.9) |  |
| **Sex** |  |  |  |
| Female | 209 (61.1) | 216 (55.4) | 0.117 |
| Male | 133 (38.9) | 174 (44.6) |  |
| **Marital Status** |  |  |  |
| Married | 248 (72.5) | 285 (73.1) | 0.779 |
| Unmarried | 68 (19.9) | 79 (20.3) |  |
| Divorced | 20 (5.8) | 17 (4.4) |  |
| Widow | 6 (1.8) | 9 (2.3) |  |
| **HTN** |  |  |  |
| Present | 103 (30.1) | 93 (23.8) | 0.056 |
| Absent | 239 (69.9) | 297 (76.2) |  |
| **BA** |  |  |  |
| Present | 31 (9.1) | 108 (27.7) | <0.001 |
| Absent | 311 (90.9) | 282 (72.3) |  |
| **IHD** |  |  |  |
| Present | 42 (12.3) | 55 (14.1) | 0.468 |
| Absent | 300 (87.7) | 335 (85.9) |  |
| **COPD** |  |  |  |
| Present | 28 (8.2) | 28 (7.2) | 0.609 |
| Absent | 314 (91.8) | 362 (92.8) |  |
| **DM** |  |  |  |
| Present | 20 (5.8) | 18 (4.6) | 0.453 |
| Absent | 322 (94.2) | 372 (95.4) |  |
| **RHS-15 Part I** | 22.96 ±8.43 | 25.44 ±6.69 | <0.001 |
| **RHS-15 Part II** | 4.42 ±1.59 | 5.39 ±1.49 | <0.001 |

HTN: Hypertension; BA: Bronchial Asthma; IHD: Ischemic Heart Disease; COPD: Chronic Obstructive Pulmonary Disease; DM: Diabetes Mellitus

*p value estimated by Chi-square test and independent samples t test where appropriate

p value <0.05 considered statistically significant

**Supplementary Table 3. RHS Scores at baseline assessment of the participants (n=732)**

| **Variable** | **RHS-15 Part I^a^** | **p-value** | **RHS-15 Part II^a^** | **p-value** |
| --- | --- | --- | --- | --- |
| **Total score** |  |  |  |  |
| Mean±SD | 24.28 ±7.65 |  | 4.94 ±1.61 |  |
| Median (Min-Max) | 24 (2 – 44) |  | 5 (0 – 10) |  |
| **Age category**  **(yrs)** |  |  |  |  |
| ≤ 30 | 23.48 ±7.75 | <0.001 | 4.91 ±1.61 | 0.655 |
| > 30 | 25.50 ±7.34 |  | 4.97 ±1.61 |  |
| **Sex** |  |  |  |  |
| Female | 24.08 ±7.62 | 0.394 | 4.99 ±1.57 | 0.364 |
| Male | 24.57 ±7.69 |  | 4.88 ±1.66 |  |
| **Marital Status** |  |  |  |  |
| Married | 24.75 ±7.47 | 0.007 | 4.97 ±1.56 | 0.470 |
| Single^b^ | 23.03 ±7.84 |  | 4.87 ±1.76 |  |
| **HTN** |  |  |  |  |
| Present | 23.51 ±7.91 | 0.096 | 4.80 ±1.60 | 0.159 |
| Absent | 24.57 ±7.54 |  | 4.99 ± 1.61 |  |
| **BA** |  |  |  |  |
| Present | 25.37 ±6.86 | 0.045 | 4.69 ±1.65 | 0.043 |
| Absent | 24.03 ±7.81 |  | 4.99 ±1.59 |  |
| **IHD** |  |  |  |  |
| Present | 25.62 ±6.01 | 0.026 | 4.98 ±1.81 | 0.744 |
| Absent | 24.08 ±7.85 |  | 4.93 ±1.58 |  |
| **COPD** |  |  |  |  |
| Present | 23.96 ±7.71 | 0.746 | 4.98 ±1.74 | 0.838 |
| Absent | 24.31 ±7.65 |  | 4.94 ±1.60 |  |
| **DM** |  |  |  |  |
| Present | 27.39 ±5.73 | 0.01 | 5.13 ±1.34 | 0.452 |
| Absent | 24.11 ±7.70 |  | 4.92 ±1.62 |  |
|  |  |  |  |  |

RHS- Refugee Health Screener; HTN: Hypertension; BA: Bronchial Asthma; IHD: Ischemic Heart Disease; COPD: Chronic Obstructive Pulmonary Disease; DM: Diabetes Mellitus

^a^RHS-15 part I consists of items 1 – 14 and part II consists of item 15 (distress thermometer).

^b^ Including unmarried, divorced and widowed.

*p value estimated by independent sample t-test

p value <0.05 considered statistically significant

**Supplementary Table 4. Mean changes in RHS-15 score between baseline and follow-up evaluation of participants (n=342)**

| **Variable** | **RHS-15 Part I^a^** | **p-value** | **RHS-15 Part II^a^** | **p-value** |
| --- | --- | --- | --- | --- |
| **Total score** |  |  |  |  |
| Mean ±SD | 23.77 ±8.78 |  | 2.48 ±2.28 |  |
| Median (Min-Max) | 24 (3 – 49) |  | 2 (-4 – 9) |  |
| **Age category** |  |  |  |  |
| ≤ 30 | 24.63 ±8.84 | 0.024 | 2.47 ±2.28 | 0.867 |
| > 30 | 22.44 ±8.55 |  | 2.51 ±2.29 |  |
| **Sex** |  |  |  |  |
| Female | 24.19 ±8.54 | 0.262 | 2.77 ±2.24 | 0.003 |
| Male | 23.09 ±9.12 |  | 2.03 ±2.29 |  |
| **Marital Status** |  |  |  |  |
| Married | 23.38 ±8.72 | 0.186 | 2.43 ±2.19 | 0.479 |
| Single^b^ | 24.78 ±8.89 |  | 2.62 ±2.53 |  |
| **HTN** |  |  |  |  |
| Present | 24.97 ±8.76 | 0.096 | 2.73 ±2.00 | 0.197 |
| Absent | 23.25 ±8.75 |  | 2.38 ±2.39 |  |
| **BA** |  |  |  |  |
| Present | 24.35 ±8.88 | 0.696 | 2.67 ±2.69 | 0.624 |
| Absent | 23.71 ±8.78 |  | 2.46 ±2.44 |  |
| **IHD** |  |  |  |  |
| Present | 20.90 ±7.47 | 0.024 | 2.52 ±2.41 | 0.908 |
| Absent | 24.17 ±8.89 |  | 2.48 ±2.27 |  |
| **COPD** |  |  |  |  |
| Present | 23.50 ±7.93 | 0.867 | 1.85 ±2.59 | 0.129 |
| Absent | 23.78 ±8.86 |  | 23.50 ±7.94 |  |
| **DM** |  |  |  |  |
| Present | 20.70 ±4.66 | 0.009 | 3.40 ±1.53 | 0.014 |
| Absent | 23.95 ±8.94 |  | 2.43 ±2.31 |  |
|  |  |  |  |  |

RHS- Refugee Health Screener; HTN: Hypertension; BA: Bronchial Asthma; IHD: Ischemic Heart Disease; COPD: Chronic Obstructive Pulmonary Disease; DM: Diabetes Mellitus

^a^RHS-15 part I consists of items 1 – 14 and part II consists of item 15 (distress thermometer).

^b^ Including unmarried, divorced and widowed.

*p value estimated by independent sample t-test

p value <0.05 considered statistically significant

**Supplementary Table 5. Participants response to COVID-19 – Impact on Quality of Life (COV19-QoL) scale.**

|  | **Completely agree** | **Disagree** | **Neither agree nor disagree** | **Agree** | **Completely agree** |
| --- | --- | --- | --- | --- | --- |
| 1. **I think my quality of life is lower than before** | - | - | - | 207 (60.5) | 135 (39.5) |
| 1. **I think my mental health has deteriorated** | - | - | 2 (0.6) | 190 (55.6) | 150 (43.9) |
| 1. **I think my physical health my deteriorate** | - | - | 1 (0.3) | 199 (58.2) | 142 (41.5) |
| 1. **I feel more tense than before** | - | - | 1 (0.3) | 148 (43.3) | 193 (56.4) |
| 1. **I feel more depressed than before** | - | - | - | 4 (56.4) | 5 (43.6) |
| 1. **I feel that my personal safety is at risk** | - | - | - | 132 (38.6) | 210 (61.4) |
